# A Novel Highly Sensitive Method for Measuring Inflammatory Neural-Derived APC Activity in Glial Cell Lines, Mouse Brain and Human CSF

**DOI:** 10.3390/ijms21072422

**Published:** 2020-03-31

**Authors:** Valery Golderman, Shany G. Gofrit, Nicola Maggio, Orna Gera, Alexandra Gerasimov, Dar Laks, Joab Chapman, Efrat Shavit-Stein

**Affiliations:** 1 Department of Neurology, Sackler Faculty of Medicine, Tel Aviv University, Tel Aviv 6997801, Israeljoabchapman@gmail.com (J.C.); 2 Department of Neurology, The Chaim Sheba Medical Center, Ramat Gan 5266202, Israel; 3 Talpiot Medical Leadership Program, The Chaim Sheba Medical Center, Ramat Gan 5266202, Israel; 4 Sagol School of Neuroscience, Tel Aviv University, Tel Aviv 6997801, Israel; 5 Department of Physical Therapy, Sackler Faculty of Medicine, Tel Aviv University, Tel Aviv 6997801, Israel; 6 Department of Physiology and Pharmacology, Sackler Faculty of Medicine, Tel Aviv University, Tel Aviv 6997801, Israel; 7 Robert and Martha Harden Chair in Mental and Neurological Diseases, Sackler Faculty of Medicine, Tel Aviv University, Tel Aviv 6997801, Israel

**Keywords:** activated protein C (APC), protease-activated receptor 1 (PAR1), endothelial protein C receptor (EPCR), thrombin

## Abstract

Background: Neural inflammation is linked to coagulation. Low levels of thrombin have a neuroprotective effect, mediated by activated protein C (APC). We describe a sensitive novel method for the measurement of APC activity at the low concentrations found in neural tissue. Methods: APC activity was measured using a fluorogenic substrate, Pyr-Pro-Arg-AMC, cleaved preferentially by APC. Selectivity was assessed using specific inhibitors and activators. APC levels were measured in human plasma, in glia cell lines, in mice brain slices following mild traumatic brain injury (mTBI) and systemic lipopolysaccharide (LPS) injection, and in cerebrospinal fluid (CSF) taken from viral meningoencephalitis patients and controls. Results: Selectivity required apixaban and alpha-naphthylsulphonylglycyl-4-amidinophenylalanine piperidine (NAPAP). APC levels were easily measurable in plasma and were significantly increased by Protac and CaCl_2_. APC activity was significantly higher in the microglial compared to astrocytic cell line and specifically lowered by LPS. Brain APC levels were higher in posterior regions and increased by mTBI and LPS. Highly elevated APC activity was measured in viral meningoencephalitis patients CSF. Conclusions: This method is selective and sensitive for the measurement of APC activity that significantly changes during inflammation in cell lines, animal models and human CSF.

## 1. Background

Neural inflammation is a core process in many pathologies, including neurodegenerative diseases [1], neoplasms [2], and nervous system reaction following trauma [3]. Inflammation and coagulation are tightly linked and modify each other in the systemic circulation [4,5,6]. The role of coagulation system proteins in neural function has been intensively studied and found to affect many processes including synaptic transmission, axonal conduction, learning, memory, and behavior [7,8,9].

Activated protein C (APC) is a serine protease participating in the coagulation cascade. Following clot formation, thrombin binds thrombomodulin (TM) and activates protein C (PC) to APC. APC binds protein S and inhibits FVa and FVIIIa which are essential for thrombin generation and thus acts as an anticoagulation factor [10]. Similar to other coagulation factors [11], APC has a role in the inflammatory response. APC inhibits pro-inflammatory cytokines, protects endothelial cells structure and function [12], and maintains normal blood pressure during sepsis [13]. APC requires two receptors for its activation and its cellular effects; the endothelial protein C receptor (EPCR) and protease-activated receptor 1 (PAR1). EPCR serves as a catalyst of PC activation and mediates APC activity [10]. When bound to EPCR, APC cleaves PAR1, and induces a positive effect in endothelial and Schwann cells [14,15]. EPCR and PAR1 are expressed in both the peripheral nervous system (PNS) [16] and the central nervous system (CNS) [17]. 

The measurement of APC activity is therefore potentially important for understanding neural function in neuroinflammation. The accepted laboratory method measures APC activity indirectly, using an in vitro activation of PC into APC by exposure to venom of southern copperhead snakes [18]. This method is expensive due to the use of snake′s venom, and only measures APC activity indirectly. Furthermore, although appropriate for the high levels of APC in the plasma (about 2.3 ng/mL) [19], this method is not sensitive enough to measure APC levels in neural tissue. 

Here we describe a novel method for APC activity measurement using the specific amino acid sequence cleaved by APC in conjugation with a fluorogenic emitting substrate. This novel method is sensitive to detect direct APC activity without the need of prior PC activation. Using this method, we measured for the first-time intrinsic APC activity in neural cell lines, mouse brain, and human cerebrospinal fluid (CSF), and were able to demonstrate significant changes in APC activity in models of neuroinflammation.

## 2. Results

### 2.1. Assay Validation

**Figure 1:**
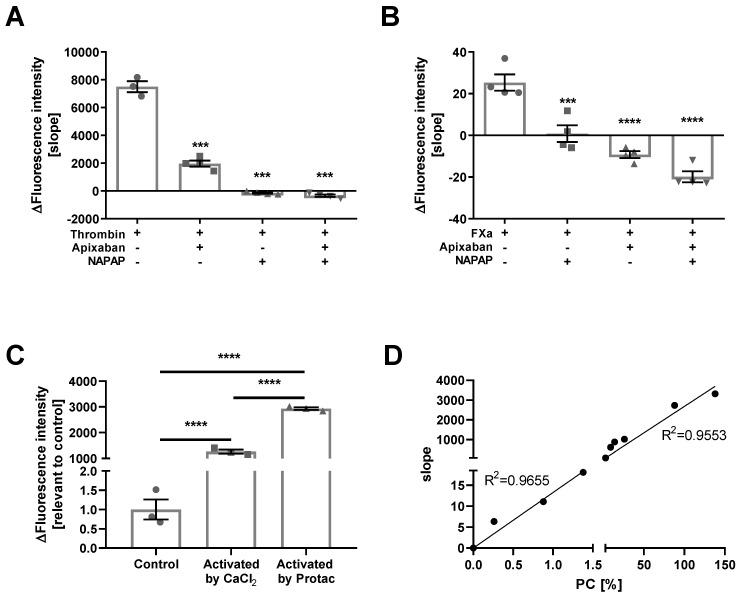
Substrate cleavage and measurement validation. (**A**) substrate cleavage by thrombin: Thrombin (50 mU) cleaves the activated protein C (APC) substrate in a non-APC manner (*n* = 3). Apixaban significantly decreases the non-APC substrate cleavage by thrombin (*n* = 4, *p* < 0.0001). alpha-naphthylsulphonylglycyl-4-amidinophenylalanine piperidine (NAPAP) and NAPAP with apixaban completely block the non-APC substrate cleavage by thrombin (*n* = 4, *p* < 0.0001). (**B**) substrate cleavage by FXa: FXa (10 mU) cleaves the APC substrate in a non-APC manner (*n* = 4). NAPAP significantly decreases the non-APC substrate cleavage by FXa (*n* = 4, *p* < 0.0005). Apixaban and apixaban with NAPAP completely block the non-APC substrate cleavage by FXa (*n* = 4, *p* < 0.0001). (**C**) APC activity in human plasma: Human citrated plasma presents low APC activity (*n* = 2), that significantly increases after activation in the presence of CaCl_2_ (*n* = 4, *p* < 0.0001) and further increases in the presence of Protac (*n* = 4, *p* < 0.0001). (**D**) standard curve: APC activity plotted versus known concentration of protein C (PC) activated by Protac, show positive linear fit (0–1.38% PC: Slope = 13.25, *R*^2^ = 0.95; 1.38–138% PC: Slope = 26.78, *R*^2^ = 0.92).

The substrate selectivity for APC activity was characterized by evaluation of its cleavage by major confounding proteases. In addition to APC, both thrombin and FXa are potentially able to cleave this sequence. Therefore, cleavage of the APC substrate by these proteases was measured. Known concentrations of commercial thrombin (50 mU) and FXa (10 mU) were used, with and without thrombin and FXa specific inhibitors, alpha-naphthylsulphonylglycyl-4-amidinophenylalanine piperidine (NAPAP), and apixaban, respectively. As can be seen in Figure 1A,B, both thrombin and FXa cleaved the substrate. The cleavage of the substrate by thrombin was fully blocked by NAPAP and partially blocked by apixaban (7500 ± 393, −140.6 ± 50.4, and 1967 ± 217 aU, respectively, *p* < 0.0001, Figure 1A). Similarly, FXa activity was fully blocked by apixaban and partially blocked by NAPAP (25.3 ± 3.9, −9.2 ± 1.6, and 0.83 ± 4 aU, respectively, *p* < 0.0005, Figure 1B). The results indicate that both thrombin and FXa cleave the substrate to a certain degree, and therefore we concluded that in order to selectively measure the APC activity, it is necessary to add NAPAP and apixaban to the assay.

Sensitivity and selectivity of the substrate for APC activity measurement was studied in human plasma. As expected, low APC activity was measured in the citrated plasma, due to the anticoagulation effect of citrate. Activation of the coagulation by CaCl_2_ caused significant increase in APC activity (1262 ± 74.73 and 1 ± 0.26 Δ in fluorescence intensity, respectively, *p* < 0.0001, Figure 1C). Further increase was achieved using a specific PC activator Protac (2931 ± 49.48 compared to control, *p* < 0.0001, Figure 1C). Next, we applied the substrate on known concentrations of PC, combined with Protac (Figure 1D). Using the substrate, we were able to detect positive APC activity, which was demonstrated by a positive slope, in samples of 138% PC (compared to normal plasma) down to 0.26% PC. The results demonstrate that the assay is sensitive to values of around 0.1% plasma PC which corresponds to a calculated concentration of approximately 5 pM. Values above this can be calculated from this calibration curve. 

### 2.2. Cell Cultures

**Figure 2:**
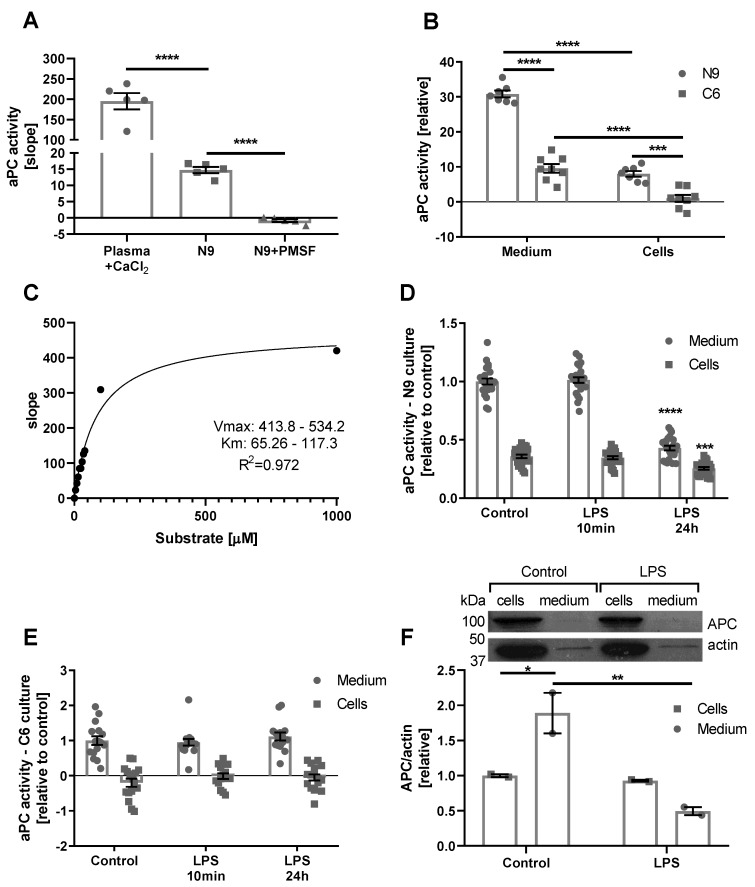
APC activity in N9 and C6 cells in normal conditions and lipopolysaccharide (LPS) model. (**A**) APC activity in activated plasma compared to N9 cells: APC activity in activated plasma (*n =* 5) is significantly higher compared to APC activity in N9 cell culture (*n =* 5, *p* < 0.0001). APC activity in N9 culture is completely inhibited following phenylmethylsulfonyl fluoride (PMSF) treatment (*n =* 5, *p* < 0.0001). (**B**) APC activity in the medium and on the cells of N9 and C6 cells: APC activity is significantly higher in the medium compared to APC activity on the cells of N9 and C6 cells (*n =* 8, *p* < 0.0001). APC activity in the medium of N9 cells is significantly higher compared to APC activity in the medium of C6 cells (*n =* 8, *p* < 0.0001). APC activity on the cells of N9 cells is significantly higher compared to APC activity on the cells of C6 cells (*n =* 8, *p* = 0.0003). (**C**) Michaelis-Menten saturation curve of the substrate: The measured activity showed a good fit to Michaelis–Menten saturation curve with *K*_m_ = 65.26–117.3 and *V*_max_ =413.8–534.2 (*R*^2^ = 0.972). (**D**) APC activity in N9 cells in LPS model: APC activity in the medium and on the cells of N9 did not changed significantly after 10 min treatment of LPS (0.1 µg/mL). Twenty-four-hour treatment of LPS significantly decreased APC activity in the medium and on the cells of N9 (*n =* 24, *p* < 0.0001 and *p* = 0.0006 respectively). (**E**) APC activity in C6 cells in LPS model: APC activity did not change significantly after short or long LPS treatment in the medium and on the cells of C6 (*n =* 16). (**F**) APC protein levels in N9 cells in LPS model: APC/actin levels are significantly higher in the medium compared to cells (*n =* 2, *p* = 0.0259) and significantly decrease in the medium following LPS treatment (*n =* 2, *p* = 0.0052).

APC activity in human plasma after activation is relatively high, and measurable even by the current routine laboratory methods. Based on our previous experience, the coagulation proteases activity in the neural tissues and cells is much lower compared to plasma, and requires a more sensitive method for detection [20,21,22,23]. In order to validate the use of the new assay in neural tissue, it was applied to N9 microglia cell culture. As expected, N9 cell culture presents significantly lower APC activity compared to CaCl_2_ activated plasma (14.75 ± 0.96 and 195.1 ± 20.04 aU, respectively, *p* < 0.0001, Figure 2A). In addition, N9 basal APC activity totally demolished with phenylmethylsulfonyl fluoride (PMSF) (−0.82 ± 0.45 aU, *p* < 0.0001, Figure 2A).

The next challenge was to examine APC activity in different cell types, and to determine whether APC is secreted into the medium or attached to the cells. Two cells types were used; N9 cells as a model for microglia, and C6 cells as a model for astrocytes [24,25]. In order to distinguish between medium and cells derived activity, the serum-free medium was transferred into empty wells 24 h after initiating the experiment. Fresh medium was added to the remaining cells and activity was measured immediately. Significantly higher APC activity was measured in the medium compared to the cells of both types (30.8 ± 0.9, 8.0 ± 0.8, 9.6 ± 1.2, and 1 ± 0.1, N9 and C6, medium versus cells, respectively, results are presented relative to APC levels measured in C6 cells, Figure 2B, *p* < 0.0001). Interestingly, APC activity in the medium and on the cells of N9 is significantly higher compared to APC activity in the medium and on the cells of C6 (*p* < 0.0001 and *p* = 0.0003). 

Next, we used N9 medium in order to determine the kinetic properties of the substrate. We applied the substrate at a rising concentrations on N9 medium and calculated from the curve the K_m_ and the *V*_max_ of the substrate (*R*^2^ = 0.972, *K*_m_ = 65.26–117.3, and *V*_max_ = 413.8–534.2, Figure 2C).

APC activity was measured in the cells in response to inflammation induced by lipopolysaccharide (LPS) (0.1 µg/mL) for short (10 min) or long duration (24 h) in the medium and on the cells. Long duration LPS treatment significantly decreased APC activity in N9 medium and cells compared to control (0.43 ± 0.02 versus 1 ± 0.03 aU in medium, 0.26 ± 0.01 versus 0.36 ± 0.01 aU in cells, *p* < 0.0001 and *p* = 0.0006, respectively). In contrast, short treatment with LPS did not affect APC activity in N9 cells (Figure 2D). In C6 cells, both short and long treatments with LPS did not change APC activity significantly (Figure 2E).

In order to verify the findings regarding APC activity in N9 cells, we conducted western blot analysis on N9 cells and medium samples following LPS treatment. As can be seen in Figure 2F, APC protein can be detected both on the cells and in the medium. Similar protein amounts were loaded for each sample but due to the high levels of albumin in the medium the apparent amount of APC seems much lower in these samples. However, both APC and actin levels are lower in the medium compared to the cells, and therefore when APC levels are corrected to actin levels, the results demonstrate significantly higher APC/actin levels in the medium compared to the cells (1.89 ± 0.29 and 1 ± 0.0195 aU, respectively, Figure 2E, *p* = 0.0259). This suggests that in the medium, most APC is present on actin containing structures such as vesicles. Following LPS treatment the APC/actin levels are significantly lower in the medium, but not on the cells, compared to controls (0.4955 ± 0.057 and 1.89 ± 0.29 aU, respectively, Figure 2E, *p* = 0.0052).

### 2.3. Mice, Healthy Control, and Mild Traumatic Brain Injury (mTBI)

**Figure 3:**
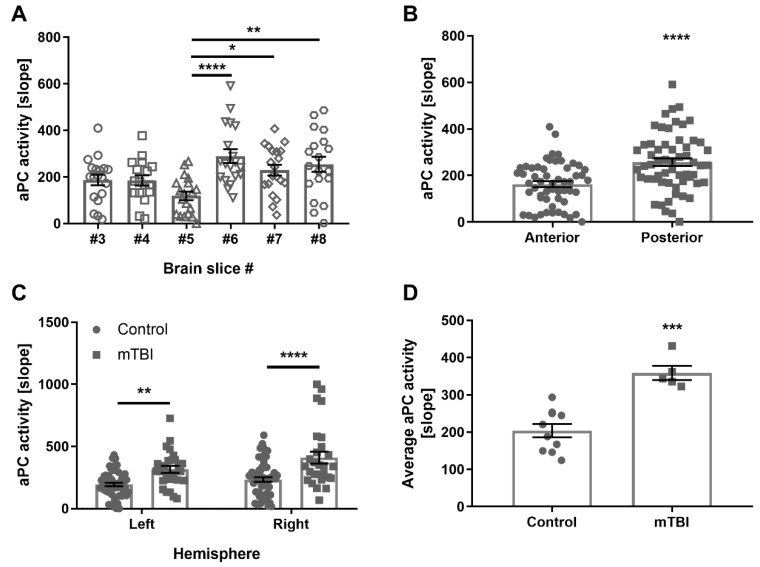
APC activity in mouse brain. (**A**) APC activity across healthy mouse brain: APC activity varies across the brain (*n =* 17–20, *p* < 0.0001). The highest activity was measured in brain slice #6, and it is significantly higher compared to brain slice #5 (*n =* 19, *p* < 0.0001). (**B**) APC activity in anterior versus posterior healthy brain slices: Posterior brain slices (#6–8) present significantly higher APC activity compared to anterior brain slices (#3–5) (*n =* 55–58, *p* < 0.0001). (**C**) APC activity in the brain 24 h after mild traumatic brain injury (mTBI): APC activity in brain slices following mTBI was significantly higher in the right and the left hemispheres, compared to control (*n =* 58, 56, and 27, respectively, *p* < 0.0001 and 0.0053, respectively). (**D**) average APC activity 24 h after mTBI: The average APC activity across the brain was significantly higher after mTBI compared to control (*n =* 5 and *n =* 10, respectively, *p* < 0.0001).

After establishing the method in vitro, APC activity was measured ex vivo. First APC activity was measured and mapped in control healthy mice brain (Figure 3A,B). A specific spatial profile of APC activity was found in the brain (*p* < 0.0001, Figure 3A). A significantly higher APC activity was found in the posterior slices of the brain compared to the anterior slices (257 ± 12.7 and 1163 ± 16.4 aU, *p* < 0.0001, Figure 3B). 

Changes in APC activity in the brain, in response to neuroinflammation associated with trauma, were evaluated. mTBI was induced in mice, followed by APC activity measurements in the brain 24 h after the injury. mTBI mice had a significantly higher APC activity in the right hemisphere of the brain compare to control (410 ± 48.3 and 233 ± 18.2 aU, respectively, *p* < 0.0001, Figure 3C). A significant elevation in APC activity was measured in the left hemisphere as well (315.6 ± 27.9 and 193.9 ± 13.6 aU, respectively, *p* = 0.0053, Figure 3C). In addition, analysis of the whole brain showed significantly higher APC activity in the brains of mTBI mice compared to control (358.9 ± 19.2 and 203.8 ± 17.9 aU, *p* < 0.0001, Figure 3D).

### 2.4. The Effect of Inflammation, LPS in Mice, and Human Inflammatory CSF

**Figure 4:**
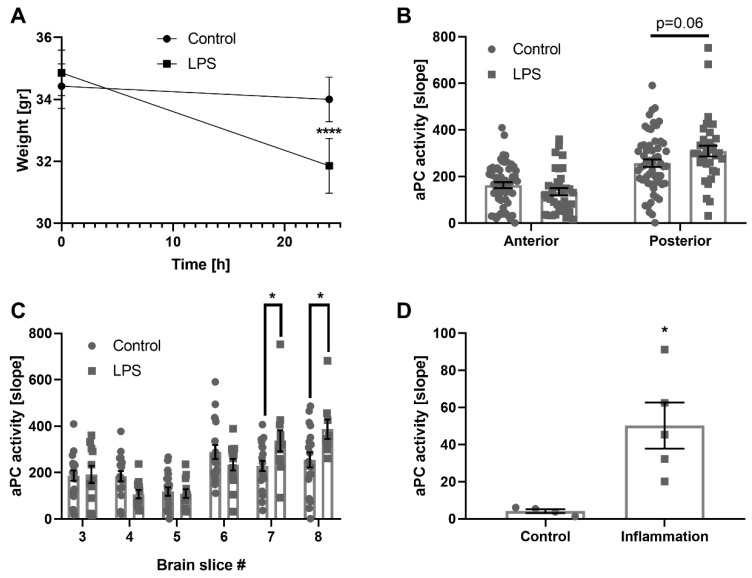
APC activity in a mice model for systemic inflammation and in human patients. (**A**) weight decline in LPS injected mice: LPS injected mice demonstrated a significant loss of weight 24 h following the injection, as a marker for the systemic inflammatory response (*n =* 7, *p* < 0.0001). (**B**) APC activity in anterior versus posterior brain slices of LPS injected mice: Posterior brain slices of systemically injected mice showed a trend towards elevated APC activity compared to posterior brain slices of control mice (*n =* 35 and 58, respectively, *p* = 0.06). (**C**) APC activity in brain slices of LPS injected mice: APC activity measurements conducted in brain slices showed significantly elevated activity in slices #7 and #8 of LPS injected mice compared to control (*n =* 9–20, *p* = 0.04 and *p* = 0.017, respectively). (**D**) elevation of APC activity in human cerebrospinal fluid (CSF) of patients suffering from systemic inflammation: CSF samples taken from patients with systemic inflammation showed significantly elevated APC activity compare to healthy controls (*n =* 5 and 4, respectively, *p* = 0.01).

Systemic inflammation was modeled using LPS. As expected, LPS injected mice lost weight compared to healthy controls 24 h following injection, as the result of general inflammation (31.8 ± 0.9 gr and 34 ± 0.7 gr, *p* < 0.0001, Figure 4A). 

APC activity was measured in the brains of LPS injected mice. A trend towards elevation was measured in the posterior brain of LPS injected mice (309.1 ± 23.4 and 257.2 ± 16.4 aU, *p* = 0.06, Figure 4B). Measurements conducted specifically in slices seven and eight in the posterior brain showed a significant APC activity elevation (337.4 ± 45.5 versus 229.2 ± 22.7 and 387.5 ± 41.8 versus 254.6 ± 32.2 aU, *p* = 0.04, *p* = 0.017, respectively, Figure 4C).

Finally, APC activity measurements were conducted in human CSF. Samples taken from viral meningoencephalitis patients showed significantly elevated APC activity compared to normal pressure hydrocephalus (NPH) controls (50.3 ± 12.4 and 4.17 ± 1.1 aU, *p* = 0.01, Figure 4D).

## 3. Discussion 

In this study, we describe a novel and sensitive method for the measurement of APC activity and validate its use in neural tissue, including cell lines, brain tissue, and CSF. We have established the appropriate conditions that enable us to distinguish APC activity from other serine proteases known to cleave the substrate. Our results indicate the need to use specific thrombin and FXa inhibitors in this APC assay. Indeed, in the presence of these inhibitors, APC activity levels in human plasma can be measured in a reliable manner. Human plasma contains significantly higher levels of PC compared to APC [26]. Addition of the potent PC activator Protac causes the expected elevation in the measured fluorescence, thus supporting the selectivity of this method, while the use of PMSF lead to the abolishment of measured fluorescence. In addition, we demonstrated the sensitivity of the standard curve in very low APC concentrations. 

APC activity assays that are available for clinical use are designed mainly for diagnosis of coagulopathies in plasma [27]. The contemporary studies on the positive effect of APC on neurons in the cellular and synaptic levels [17,28] require a more sensitive assay. Although activity levels measured in calcium activated plasma are higher than in N9 cells, the difference is only about one order of magnitude. This falls well within the sensitivity of the assay, supporting its use for the detection of APC activity in neural cells. Here, this method was used for the first time in neural tissue. Using a range of substrate concentrations, we were able to fit a Michaelis–Menten saturation curve, allowing for kinetic calculations. We demonstrate for the first time that N9 microglia cells and C6 glioma cells have intrinsic APC activity. Interestingly, APC activity varies between those cell types being higher in N9 microglia cells [29], compared to C6 astrocytic cells. Microglia are immune cells, taking part in inflammatory responses and neuroregenerative changes [30]. APC has been shown to induce neuroregeneration [31] but the possibility that it is produced locally by the neural tissue was not studied before. Our results demonstrating elevated APC activity in microglia cells may suggest a possible involvement of this pathway in microglial induced neuroregeneration. We further localized APC activity to their surrounding medium. APC activity levels in both cell types are higher in the medium, suggesting the secretion of APC, as a soluble molecule or as part of an extracellular vesicle. This may be part of a complex interaction process with neighboring cells [26,32]. The presence of APC in the cells and the surrounding medium is further supported by western blots (Figure 2F). Results were standardized according to actin rather than general protein concentration due to the abundance of albumin in the medium. Interestingly, significance levels of actin were measured in the medium as well, which suggests vesicular exocytosis as a possible mechanism for APC secretion. 

We also were able to utilize this method for measuring APC activity ex vivo. Measurements of APC in mice brains show elevated APC activity levels in the posterior brain sections. This may be due to high concentration of microglia in these sections [33]. An expected elevation of APC activity was measured following mTBI in mice. This probably represents the inflammatory response initiated by the trauma [34] and the involvement of microglia in this pathophysiology. As mentioned above, APC levels were elevated in the hemisphere contralateral to the injury as well, suggesting the systemic effect of mTBI on the brain. It was previously shown that brain injury leads to microparticles release by the microglia, as a neuroinflammatory response [35]. Our in vitro results, showing APC secretion by microglia cells, together with our ex vivo observations, suggest that these microparticles may be the source of the elevated APC activity in the brain following mTBI. 

Systemic injection of LPS creates a general inflammatory response, including brain involvement. Elevated brain thrombin activity, together with PC and EPCR upregulation, following LPS injection has been previously described [36]. Thus, we expected to measure changes in APC activity following LPS injection. In contrast to the results in the mTBI model, elevation of APC activity in the LPS model is prominent mostly in the posterior brain. High concentration of microglia cells in the posterior parts of the brain and our findings, suggest that LPS leads to elevated APC activity levels through local microglia activation. The ex vivo brain slices, as well as the mTBI and the LPS, are all murine models. APC is relatively conserved across different species. Cleavage of chromogenic substrate was previously shown to be almost identical in both human and mice [37]. Since the cleavage site amino acids sequence used in the present work was not changed, results obtained using murine models may represent the results expected in humans.

Finally, we utilized the method for measuring APC activity in human samples. We assumed that changes in brain APC activity, following neuroinflammation, would be measurable in the CSF. As expected, APC activity was significantly higher in CSF taken from patients with viral meningoencephalitis, compared to NPH controls. These results demonstrate that this novel method can be used for diagnosis and drug efficacy evaluation in human subjects. 

It is important to consider the opposite responses of APC activity were seen in vitro and ex vivo in the different inflammation models in this study. In the N9 cell line LPS model in vitro, APC activity was decreased after 24 h of exposure while, in contrast, elevated APC activity measured in the brain following systemic LPS administration and mTBI in mice and in acute inflammation in human CSF. The inflammatory response consists of an early phase and a recovery phase. Inflammation resolving molecules is down-regulated in the early phase and up-regulated in the recovery phase [38]. APC has an anti-inflammatory effect, through tissue factor (TF), thrombin inhibition, and down-regulation of pro-inflammatory cytokines and chemokines [39]. Accordingly, one may assume that APC will act as an inflammation resolving molecule and therefore its activity will vary over the inflammatory response. Our models probably represent two different points in time of the inflammatory response.

In conclusion, in this report we describe a novel method for the measurement of APC activity. This sensitive tool may be of value in the study of the cellular effect of APC and its effect on neural function and survival. Accurate measurements of APC may be used in the evaluation of new pharmacological intervention in the EPCR/PAR1 pathway. 

## 4. Methods 

### 4.1. Cell Culture

C6 rat glioma cells (purchased from ATCC, CCL-107) and N9 mouse microglia cells (a generous gift from Professor Reuven Stein, Department of Neurobiology, George S. Wise Faculty of Life Sciences, Tel Aviv University, Tel Aviv, Israel) were grown in Dulbecco′s modified Eagle′s medium (DMEM; Bet Haemek, Biological Industries, Kibbutz Beit-Haemek, Israel) supplemented with 10% fetal bovine serum (Bet Haemek, Biological Industries, Kibbutz Beit-Haemek, Israel), 1% l-Glutamine (Bet Haemek, Biological Industries, Kibbutz Beit-Haemek, Israel), and 0.1% penicillin and streptomycin (Bet Haemek, Biological Industries, Kibbutz Beit-Haemek, Israel). The cells were grown in a 37 °C and 5% CO_2_-humidified atmosphere.

### 4.2. Animals

Adult ICR male mice (Envigo, Jerusalem, Israel) were housed in standard conditions and fed standard diet and water available ad libitum. Ambient temperature was set to 22 °C to 23 °C with day/night light control. The protocols of this study were approved by the Chaim Sheba Medical Center Committee on the Use and Care of Animals (1084-17, 1000/15) according to the ARRIVE Guidelines.

### 4.3. Mild Traumatic Brain Injury (mTBI) Induction 

Mice were anesthetized using isoflurane. The mouse head was placed on a sponge immobilization board and a 50 gr metal weight was dropped down an 80 cm-high metal tube placed over the head of the mouse on the right anterolateral side. Control mice were anesthetized only. 24 h following mTBI induction mice were sacrificed and the brain was removed for the APC activity assay.

### 4.4. Mice Lipopolysaccharide (LPS) Treatment 

Mice were weighed before LPS induction. Mice were injected intraperitoneal (IP) with LPS, a component of the bacterial wall (*Escherichia coli* 0111:B4, Sigma-Aldrich, catalog no. L4130, Jerusalem, Israel) 1 mg/kg, diluted in saline. Twenty-four hours following injection, mice were weighted and sacrificed. Brains were removed and prepared as described below. 

### 4.5. Human Patients 

CSF samples were collected from viral meningoencephalitis patients and normal pressure hydrocephalus patients by a lumbar puncture, and were kept in −80 °C until use. The procedure was approved by the ethical committee of the Chaim Sheba Medical Center (4245-17-SMC), and patients provided written informed consent. 

### 4.6. APC Activity Assay

APC activity was assessed using a fluorogenic substrate synthesized on order by our specifications (Pyr-Pro-Arg-AMC, 20 µM, GL Biochem Shanghai Ltd., Minhang district, Shanghai, China). The reactions were carried out in black 96-well microplates (Greiner, catalog no. 655096 for cells and Nunc, catalog no. 237108 for tissue). The reactions with commercial thrombin, FXa, plasma, and brain tissue were performed in Tris buffer (in mM: 150 NaCl, 1 CaCl_2_, 50 Tris-HCl: pH 8.0). The cleavage of the substrate was measured at 37 °C every 2 min over 25 cycles (excitation 360 ± 9 nm and emission 465 ± 20 nm) using microplate reader (Tecan; infinite 200; Mannedorf, Switzerland). The activity was calculated as the linear increase of fluorescence intensity over time.

### 4.7. Thrombin, FXa, Plasma, and PC

Bovine thrombin (50 mU, Sigma, T4648, Jerusalem, Israel), bovine FXa (1 mU, Haematologic Technologies, BCXA-1060, Essex Junction, VT, USA) were used. Pooled human citrated plasma from healthy donors was purchased from Instrumentation Laboratory (normal control Assayed, 0020003110, Bedford, MA, USA) and reconstituted using standard laboratory methods. Reference standard PC (138%, 88%, and 26%) were used (Technoclone, 5341013, Vienna, Austria).

### 4.8. Experimental Design

#### 4.8.1. Thrombin/FXa/Plasma

Bovine thrombin, bovine FXa or human plasma were added to the wells (final volume of 100 µL). For plasma activation, calcium chloride (CaCl_2_) was added to the plasma (final concentration: 25 mM) and incubated for 10 min in 37 °C. In the relevant wells, the samples were incubated with Protac (Technoclone, 5346212) for 5 min in order to activate PC. Substrate with the inhibitors NAPAP (1 µM, Santa Cruz, sc-208083, Dallas, TX, USA) and apixaban (1 µM, Selleckchem, S1593, Houston, TX, USA) was added and the fluorescence was measured.

#### 4.8.2. Cells

Cells were seeded (1 × 10^5^ cells/mL, 200 µL/well) and allowed to attach for 24 h. The medium was then replaced by serum free medium (DMEM, 100 µL). LPS was added (0.1 µg/mL, O111:B4, Sigma, L4130) at the beginning, or 10 min before the end of the 24 h incubation period (long and short treatments, respectively). In the relevant wells, the samples were incubated with PMSF (20 mM) for 10 min. In order to measure APC activity in the medium and on the cells, the medium was transferred to empty wells and fresh medium (with the appropriate treatment) was added to the cells. Substrate with inhibitors (NAPAP, apixaban) was added immediately and the fluorescence was measured.

For substrate kinetics measurements, cells were seeded (1 × 10^6^ cells/flask) and allowed to attach for 24 h. The medium was then replaced by serum free media and following 24 h incubation 100 µL from this medium were transferred to the wells. Range of substrate concentrations (0–1000µM), with inhibitors (NAPAP, apixaban), was added to the medium and the fluorescence was measured.

#### 4.8.3. Brain Tissue

Mice were sacrificed by lethal phenobarbital injection (CTS Chemical Industries Ltd., Kiryat Malakhi, Israel). The brain was rapidly removed and placed in a steel brain matrix (1 mm, Coronal, Stoelting, IL, USA) on ice. Brains were cut in the sagittal plane to separate right and left hemispheres. Next, brains were cut in the coronal plane into 1 mm slices. Left and right sections of slices three to eight were placed in the wells and the microplate was kept on ice until the addition of the substrate. Substrate containing inhibitors (NAPAP, apixaban) was added and the fluorescence was measured.

### 4.9. Western Blot Analysis

#### 4.9.1. Samples Preparation

Cells were seeded (1 × 10^6^ cells/flask) and allowed to attach for 24 h. The medium was then replaced by serum free medium and following 24 h the samples were collected on ice as following: First the medium was transferred and proteases and phosphatases inhibitors (protease inhibitors cocktail (1:100, 539134, Calbiochem, San Diego, CA, USA), PMSF (2 mM, P7626, Sigma) and sodium orthovandate (1 mM, S6508, Sigma)) were added to the medium. The cells were washed with cold PBS, RIPA lysis buffer (in mM: 50 Tris-HCl pH 8.0, 150 NaCl, 1% NP-40, 0.5% Sodium Deoxycholate, and 0.1% SDS) containing proteases and phosphatases inhibitors was added and the cells were scraped and collected. The collected medium was transferred to centrifugal filter device 10 K (UFC901008, Merck–Millipore, Molsheim, France), centrifuged (4000 *g*, 20 min), and the concentrated sample was collected for further analysis. The cells incubated on ice for 10 min, centrifuged (16000 *g*, 20 min), and the supernatant was collected for further analysis.

#### 4.9.2. Protein Detection

Protein concentration in the samples was evaluated using bicinchoninic acid protein assay (BCA, Pierce, 23225, Qiryat Shmona, Israel) and calibrated using known dilutions of bovine serum albumin (BSA). Proteins in the samples (20 μg total protein) were separated by polyacrylamide gel electrophoresis and transferred onto nitrocellulose membranes for Western blot analysis as described previously [40]. Membranes were incubated with primary rabbit anti-APC antibody (1:400, MBS355196, MyBioSource, Ontario, Canada) over night at 4 °C. Membrane were then washed with Tris-buffered saline and 0.1% Tween 20 (TBST) and incubated with horseradish peroxidase-conjugated goat anti-mouse antibody (1:10,000, Jackson Immunoresearch Laboratories, West Grove, PA, USA) in room temperature for 1 h. Peroxidase-based enhanced chemiluminescence (ECL) method was used for protein bands detection. ImageJ, a java-based image processing software was used for protein bands density analysis (Bethesda, MD, USA). 

### 4.10. Statistics 

Statistical analyses and graphs were conducted using GraphPad Prism (version 7.00 for Windows, GraphPad Software, La Jolla, CA, USA, www.graphpad.com). Paired *t*-tests, one-way ANOVA, and two-way ANOVA followed by a post hoc test were applied on normally distributed data sets. One-way ANOVA was followed by either Dunnett’s or Tukey’s post hoc analyses. Two-way ANOVA was followed by Sidak’s post hoc analysis. Results of post analyses are presented only when ANOVA results show statistical significance. Mann Whitney test was applied on non-normal distributed data sets. Results are expressed as mean ± SEM, *p* values < 0.05 were considered significant. 

## Abbreviations

Activated protein C	APC
Thrombomodulin	TM
Mild traumatic brain injury	mTBI
Cerebrospinal fluid	CSF
Lipopolysaccharide	LPS
Endothelial protein C receptor	EPCR
Protease activator receptor 1	PAR1
Peripheral nervous system	PNS
Central nervous system	CNS
Normal pressure hydrocephalus	NPH
Phenylmethylsulfonyl fluoride	PMSF
Alpha-naphthylsulphonylglycyl-4-amidinophenylalanine piperidine	NAPAP
